# Combining qualitative and quantitative operational research methods to inform quality improvement in pathways that span multiple settings

**DOI:** 10.1136/bmjqs-2016-005636

**Published:** 2017-01-06

**Authors:** Sonya Crowe, Katherine Brown, Jenifer Tregay, Jo Wray, Rachel Knowles, Deborah A Ridout, Catherine Bull, Martin Utley

**Affiliations:** 1 Clinical Operational Research Unit, University College London, London, UK; 2 Great Ormond Street Hospital NHS Foundation Trust, London, UK; 3 Population, Policy and Practice Programme, UCL Institute of Child Health, London, UK

**Keywords:** Quality improvement methodologies, Healthcare quality improvement, Implementation science

## Abstract

**Background:**

Improving integration and continuity of care across sectors within resource constraints is a priority in many health systems. Qualitative operational research methods of problem structuring have been used to address quality improvement in services involving multiple sectors but not in combination with quantitative operational research methods that enable targeting of interventions according to patient risk. We aimed to combine these methods to augment and inform an improvement initiative concerning infants with congenital heart disease (CHD) whose complex care pathway spans multiple sectors.

**Methods:**

Soft systems methodology was used to consider systematically changes to services from the perspectives of community, primary, secondary and tertiary care professionals and a patient group, incorporating relevant evidence. Classification and regression tree (CART) analysis of national audit datasets was conducted along with data visualisation designed to inform service improvement within the context of limited resources.

**Results:**

A ‘Rich Picture’ was developed capturing the main features of services for infants with CHD pertinent to service improvement. This was used, along with a graphical summary of the CART analysis, to guide discussions about targeting interventions at specific patient risk groups. Agreement was reached across representatives of relevant health professions and patients on a coherent set of targeted recommendations for quality improvement. These fed into national decisions about service provision and commissioning.

**Conclusions:**

When tackling complex problems in service provision across multiple settings, it is important to acknowledge and work with multiple perspectives systematically and to consider targeting service improvements in response to confined resources. Our research demonstrates that applying a combination of qualitative and quantitative operational research methods is one approach to doing so that warrants further consideration.

## Introduction

Improving continuity^1–8^ and integration^9–12^ of care across sectors is a priority in many health systems. Increasingly, patients are discharged to community care as early as clinically appropriate to alleviate pressure on hospitals and in response to patient preferences for receiving care closer to home.^13^ ^14^ However, it is challenging to design and assure the quality of services that are delivered across multiple organisations, not least because responsibility is dispersed among health professional groups that have different priorities and perspectives.

Literature on the development of complex interventions^15–17^ and guidance on service design^18^ stress the importance of evidence-informed approaches to concentrate attention and scarce resources on changes most likely to be effective.^19–21^ Yet, the evidence to support service change may be incomplete and comprise disparate quantitative and qualitative data as well as tacit knowledge.^22–26^ Formal group consensus methods such as Delphi and nominal group technique have been used extensively to incorporate the collective tacit knowledge of experts in the formulation of clinical practice guidelines and selection of outcome measures and quality indicators.^27–30^ However, such approaches are designed to obtain consensus regarding specific issues and are less readily applicable in group processes aiming to characterise complex service problems and to reach decisions about how to tackle these in ways that explicitly accommodate diverse perspectives and motivations.^31^

Operational research (OR) approaches to structured group decision-making are distinctive in their use of formal models to represent the problem in a manner that is amenable to analysis and manipulation.^32^ Formal models need not be quantitative, and a class of qualitative problem-structuring methods within the ‘soft’ (interpretivist) OR paradigm exists to help groups explore and address complex problems.^33^ These approaches permit pragmatic partial or local improvements to be agreed without requiring consensus among different interests on an overall solution.^34^ They include decision-conferencing,^35^ facilitative modelling,^36^ cognitive mapping,^37^ strategic options development and analysis,^37^ and strategic choice approach.^38^ The most relevant for system redesign is soft systems methodology, designed to tackle complex issues through systematic learning about the problem, decision processes and levers of change.^39^ Problem-structuring methods have predominantly been used to address issues in single organisations rather than those that span organisations, the latter being more challenging given the diffuse decision-making and greater diversity of working practices and goals.^40^ Recent notable exceptions include the use of soft systems methodology to assess existing provision and identify improvement strategies for children's mental health services^41^ and stroke rehabilitation.^42^

Quantitative techniques within the ‘hard’ (positivist) OR paradigm (eg, queuing theory, optimisation, simulation and statistical analysis^43–45^) support decision-making by using reductionist models to quantify the potential impact of proposed actions. Although the purpose of quantitative analyses within OR is to inform decisions and improvement rather than to generate knowledge, the implementation of quantitative OR methods is low in healthcare.^44^ ^46–48^ One possible explanation is that reductionist approaches used in isolation capture insufficient understanding of the nature and context of complex issues to give relevant and practical findings and fail to secure buy-in and acceptance from stakeholders.^49^ It has been argued that combining quantitative and qualitative OR methods can enhance the likelihood of beneficial adoption.^49^ ^50^ Studies using qualitative and quantitative OR methods in healthcare applications have predominantly taken the form of problem-structuring followed by simulation modelling,^36^ ^51^ ^52^ and have largely been confined to single organisation settings.^53^

We set out to combine soft systems methodology and quantitative OR methods to facilitate and inform an improvement initiative concerning a complex care pathway spanning multiple sectors. Our approach was designed to support a stakeholder group in developing and agreeing targeted, evidence-informed recommendations for service improvement within a case study provided by an existing research project. Following Eden^54^ and others, we judged the effectiveness of our approach by the material impact it had on the process of producing the recommendations rather than the eventual implementation or system outcomes in the much longer term.^55^ We conclude by reflecting on the potential for complementary qualitative and quantitative OR methods to augment service design and quality improvement work involving services that span multiple sectors and professions.

## Methods

### Case study setting

Our case study setting was a UK research project investigating the outcomes and support services for infants discharged following intervention for congenital heart disease (CHD) using qualitative data regarding the experiences of families, health professionals and helpline staff and quantitative national audit data (see box 1 for further details).^56–61^ OR did not feature in the original research design, but was added to inform the process of analysing and using the data obtained to develop recommendations for improving services.

Box 1 Services for congenital heart disease and the programme of research in our case study Services for congenital heart disease in the UK

In the UK, paediatric cardiac surgery for congenital heart disease (CHD) is commissioned nationally and delivered in specialist tertiary centres. Following surgical or catheter intervention in these centres, patients are discharged home, sometimes via their secondary care hospital team. Children continue to receive follow-up outpatient care at the tertiary centre, and potentially in outreach clinics locally. Some may receive home monitoring facilitated by community nurses and/or specialist cardiac nurses at the tertiary centre. All infants and their parents have access to a local general practitioner (GP) and a health visitor (nurses or midwives with additional training as specialist community public health nurses). The local services are primarily commissioned through Clinical Commissioning Groups (CCGs).

The programme of research in our case study

The number and complexity of infants with CHD surviving intervention and requiring care in the community following discharge from the specialist surgical centres are increasing.^62^ A 2-year multidisciplinary programme of research was conducted in response to concerns over mortality levels in this patient population, variability in the provision of services at and following discharge, and barriers to care experienced by some families. It included conducting:

- *A systematic review* of potential risk factors for unexpected deaths and emergency readmissions following hospital discharge;^56^
- *Statistical analyses* of national CHD and paediatric intensive care audit datasets to develop a risk model of death or emergency readmission to intensive care in the year following hospital discharge;^57^
- *Interviews* regarding care at or following discharge with: 20 parents of children who had either died following discharge or had been readmitted as an emergency to intensive care; 38 health professionals (from primary, secondary and tertiary organisations) and 10 helpline staff based at charitable organisations;^58^ ^60^
- *An online discussion forum* with parents of children with CHD regarding their experiences accessing support at or following discharge from a specialist hospital.

A key aim of the research was to use the information being generated to identify ways to improve the provision of care and support for this patient population in the discharge and postdischarge period.^61^

### Methodological approach: combining qualitative and quantitative OR methods

Our approach was tailored to the multiple aspects of the problem and the multiple phases of OR involvement.^50^ ^63^ Services for infants following discharge involve health professionals from many different organisations and backgrounds, so it was important to understand and incorporate a diversity of perspectives in order to enhance the feasibility and acceptability of the findings. Soft systems methodology (SSM)^64^ ^65^ was deemed the most appropriate choice of problem-structuring method, given its focus on acknowledging and engaging multiple perspectives in developing interventions.^33^ ^66^ It was also apparent that resource availability would constrain improvements, and so we explored how recommendations might be targeted at specific patient groups. Classification and regression tree (CART) analysis^67^ was selected as an appropriate quantitative OR technique for identifying patient groups with different risk profiles.

These complementary OR methods were interwoven,^53^ with the initial focus on designing and conducting data analysis to inform subsequent stakeholder decision-making (drawing on SSM to analyse qualitative data and CART to analyse quantitative data). SSM also provided an overarching framework for the stakeholder group's decision-making process, including a facilitated workshop at the end of the project. The CART analysis informed the participative decision-making in the final workshop through a graphical representation designed for that purpose.

### Qualitative OR methods

In our case study, the key stakeholders were as follows:

An *Advisory Group* comprising patient group representatives; health professionals from three tertiary cardiac centres; representatives from primary and secondary care; academics in psychology, statistics, epidemiology and OR;

A *Working Group* convened to propose recommendations for service improvements, which comprised selected members of the Advisory Group and invited additional representatives from the tertiary, community and charitable sectors;

*Participants of a facilitated workshop* for parents of infants who had undergone intervention and subsequently either died after discharge or required emergency readmission to intensive care.

Box 2 outlines the principles by which problematic situations are conceived and approached in SSM, as described originally by Checkland and Poulter.^65^

Box 2 **The principles of soft systems methodology (SSM)**

Adapted from Checkland and Poulter.^65^

Principle 1 SSM is concerned with real-world ‘problematic situations’, that is, real situations which someone thinks need attention and action.

Principle 2 All thinking and talking about problematic situations will be conditioned by the worldviews of the people doing the thinking and talking.

Principle 3 Every problematic situation will contain people trying to act *purposefully*, with intent. This means that *models of purposeful activity*, in the form of systems models built to express a particular worldview, can be used as *devices* to explore the qualities and characteristics of any problematic situation.

Principle 4 Discussion and debate about such a situation can be *structured* by using the models in Principle 3 as a source of questions to ask about the situation.

Principle 5 Acting to improve a problematic situation entails finding, in the course of the discussion/debate in Principle 4, accommodations among different worldviews, that is, finding a version of the situation that people with different worldviews can live with.

Principle 6 The inquiry created by Principles 1–5 is, in principle, a never-ending process of learning.

Principle 7 Explicit organisation of the process which embodies Principles 1–6 enables and embodies conscious critical reflection about both the situation itself and also the thinking about it.

These principles underpinned the four defining actions of the SSM approach described below as undertaken in our case study. In line with standard SSM practice, these actions were conducted iteratively in a non-linear process.

#### SSM action 1—finding out about the situation (using Rich Pictures)

We first developed an account of the problem in the form of a ‘Rich Picture’ that captured key features of the services (eg, the people, processes, places, relationships and viewpoints involved), perceived issues (eg, barriers to accessing care) and the characteristics of possible improvements from a ‘systems thinking’ perspective.^68^ Our initial Rich Picture was based on narratives from interviews conducted in the case study research project (see box 1). We then added information relating to risk factors and important features of heterogeneity in this patient population that emerged from the systematic review and analyses of national audit datasets (see box 1). This version of the Rich Picture was used within a facilitated workshop of interviewed parents to share findings and explore potential service improvements from their perspectives. The Rich Picture was then augmented with learning from this workshop as well as evidence-informed suggestions for service improvements generated through a facilitated process by the Advisory Group. At this stage, prompts for guiding further discussions about quality improvement were explicitly added (eg, ‘which patients are prioritised for each intervention?’). The augmented Rich Picture was used to familiarise members of the Working Group with the features of the problem being addressed, and additional contributions from their perspectives were captured.

Another component of action 1 was the familiarisation of the operational researcher conducting the SSM (SC) with the problem setting in advance of, and continuing alongside, the group activity of developing the Rich Picture.

#### SSM action 2—building conceptual models (Root Definitions and Activity Diagrams)

The process of generating a Rich Picture highlighted the different motivations, priorities and constraints of health professionals across the organisations involved. These constitute different ‘worldviews’, which are important to articulate as part of the process of learning about and improving the situation. This involved constructing a structured statement called a ‘Root Definition’ for each of the worldviews identified from the interviews with health professionals. We used the SSM mnemonic ‘CATWOE’ as a way of systematically including information in each Root Definition about:

*C*ustomers (eg, infants undergoing cardiac intervention);

*A*ctors (eg, health professionals);

*T*ransformation process (eg, clinical content of the service);

*W*orldview;

*O*wners (eg, national specialist commissioning);

*E*nvironmental constraints (eg, shortage of time for staff).

Conceptual models called ‘Activity Diagrams’ were also constructed, each comprising a set of linked purposeful activities that encapsulate a particular worldview. Such models are not intended to describe completely the real world (since each is based only on a single worldview), but rather are devices to explore potential improvements in an organised way. Activity Diagrams for each worldview were initially constructed using the Root Definitions and narratives expressed in health professional interviews and then checked for face validity with the members of the Working Group.

#### SSM actions 3 and 4—using conceptual models to question the situation and defining action to improve

An operational researcher (SC) used the conceptual models to develop questions exploring where changes to services could be made and what the implications might be from different perspectives. Each of the conceptual models and their related questions were then discussed individually with a relevant health professional from the expert Working Group. In a parallel process, draft evidence-informed service improvements were generated by constructing a hyperframework of qualitative analyses, identifying archetypal service problems, and linking these to candidate recommendations using data generated in the research project (as described in ref. 61). As part of the SSM approach, a final workshop with the entire Working Group was convened in which the operational researcher facilitated a structured discussion about the feasibility and acceptability of the drafted service improvements from different worldviews, informed by learning captured in the Rich Picture, conceptual models and individual conversations about potentially conflicting viewpoints or processes. The aim of the workshop was to agree upon a set of recommendations for service improvements with broad stakeholder endorsement.

### Quantitative OR methods

The research project in our case study included the development of a risk model for late adverse events (death or emergency readmission to intensive care in the year following hospital discharge) using linked national datasets.^53^ This was effective in generating knowledge about patient-level factors independently associated with these outcomes, but did not provide concrete information to guide the clinical community in improving services for this patient population. We therefore used complementary CART analysis designed to identify subgroups of patients that might benefit from different interventions because of the differing nature and scale of their risk. This resulted in the division of the population into six groups, each defined by a combination of risk factors known at the point of discharge such as non-cardiac comorbidity, ‘high-risk’ primary cardiac diagnosis and long length of stay in hospital. Details regarding the methods of analysis and the characteristics of these groups are presented elsewhere^57^; here, we focus on the process by which it fed into the development of the recommendations.

Specifically, a visual representation of the analysis was designed to illustrate, in an intuitive and engaging manner, the relative size of the patient groups alongside their risks of adverse outcome to inform the Working Group's thinking about how limited resources might be targeted most effectively. This visual representation was integrated within the SSM approach at the final workshop, in which the operational researcher facilitated the Working Group in considering the visual aid, the characteristics of the patient groups, the nature of the potential service improvements and findings from qualitative research where this provided additional information not captured in routinely collected national audit data.

### Study ethics

The collection and analysis of quantitative and interview data and the generation of recommendations were conducted as part of a research project funded by the National Institute for Health Research, which had Research Ethics Committee approval. All interviewees provided informed consent.

## Results

### Qualitative OR

**Figure 1:**
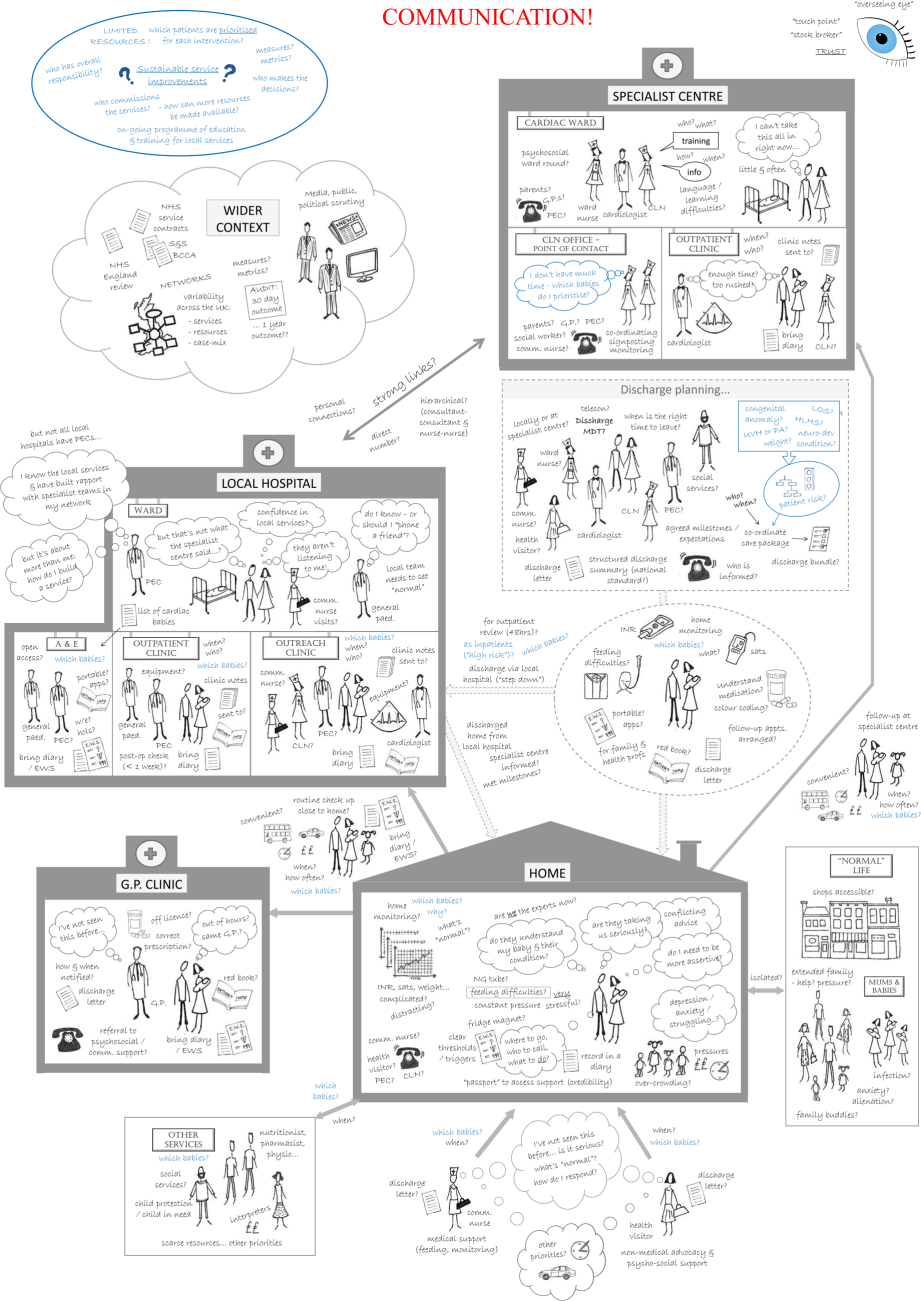
Rich Picture generated in case study. The Rich Picture that was generated using a combination of interview data,^53–55^ data analysis^57^ and further engagement with stakeholders, including families and health professionals from different health service settings. PEC, paediatrician with expertise in cardiology; GPs, general practitioners; CLN, cardiac liaison nurse; MDT, multidisciplinary team; LOS, length of stay; HLHS, hypoplastic left heart syndrome; UVH, univentricular heart; PA, pulmonary atresia; INR, international normalised ratio (blood-monitoring machine); EWS, early warning system; S&S, safe and sustainable review; BCCA, British Congenital Cardiac Association.

Figure 1 shows the Rich Picture, which was printed as posters approximately 85×60 cm. It captures key features of the entire patient journey including the different sectors that provide services, the health professionals involved and the interactions and flows of information between them. The family appear with their baby in all of the settings, and as they move through their journey, they express different concerns and issues that need addressing. Blue text highlights any information relating to risk factors, important features of heterogeneity in this patient population and prompts for guiding further discussions about quality improvement. The word ‘communication’ is highlighted in large red letters to reflect the widely held view that poor communication between health professionals and with families was a major system-wide problem. The ‘overseeing eye’ in the top right corner reflects one of the key concerns of the families, namely the lack of a single point of contact with responsibility for coordinating the entire care journey, which they identified as an important area for improvement. A separate cloud bubble shows aspects of the ‘wider context’ identified by stakeholders as affecting the situation and potential improvements, such as a national review of services for CHD being conducted at the time.

**Figure 2:**
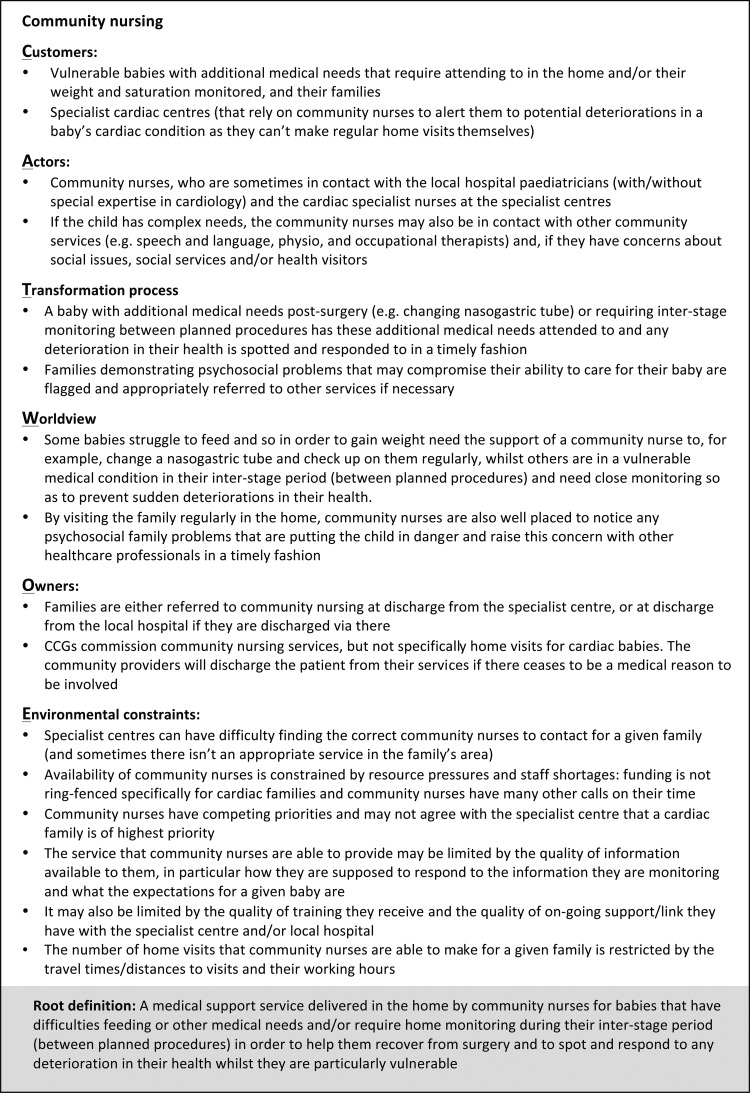
CATWOE and Root Definition for community nursing. These examples of a CATWOE and Root Definition are from the perspective of community nursing (one of the five ‘world views’ we explored) and were developed from the narratives expressed in interviews with community nurses. These underpin the Activity Diagram shown in figure 3. CCG, Clinical Commissioning Group. CATWOE, mnemonic for systematically including information about customers, actors, transformation process, worldview, owners and environmental constraints.

**Figure 3:**
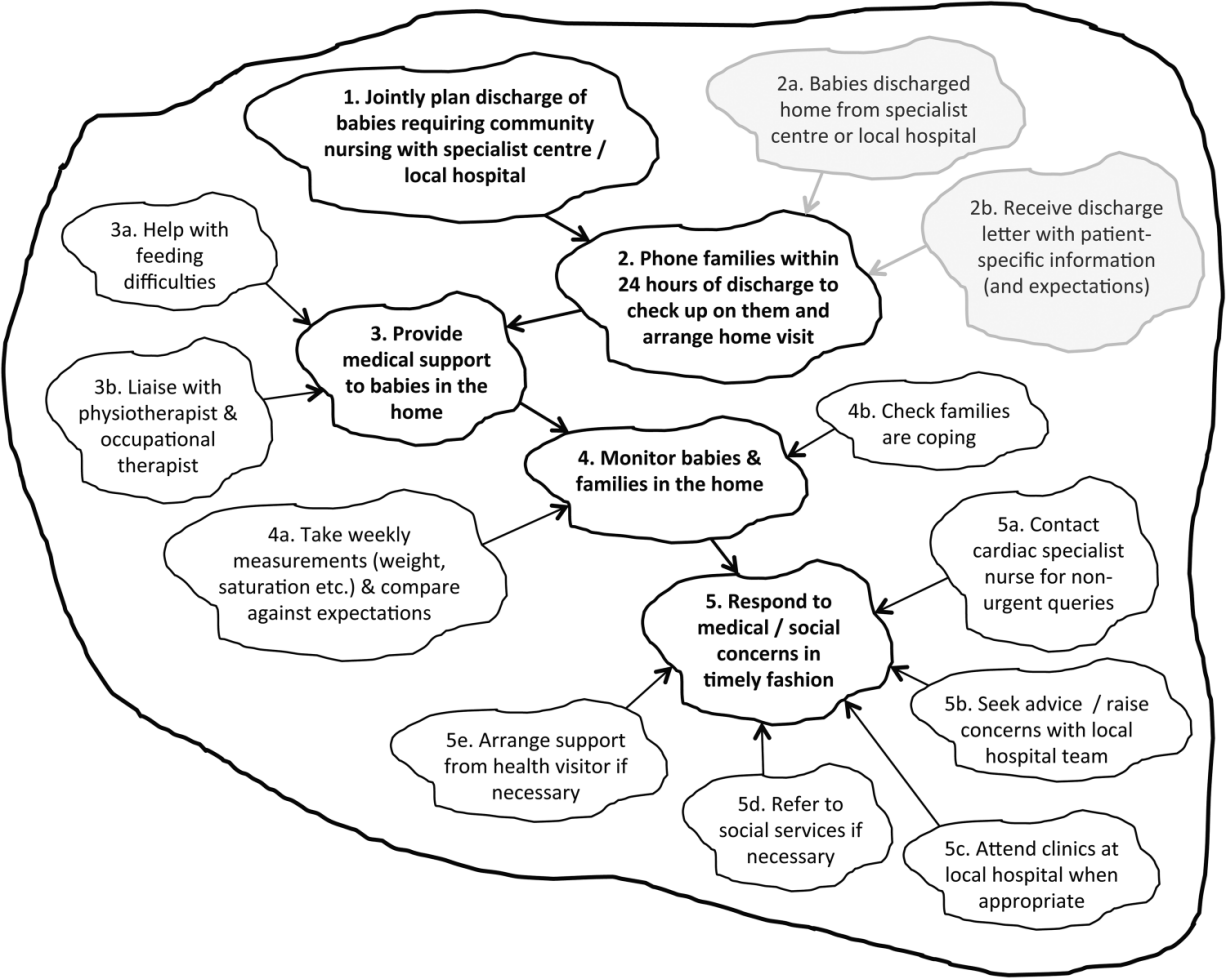
Activity Diagram for community nursing. This shows the linked set of purposeful activities required to deliver the community nursing services described in the Root Definition (figure 2). The five main activities are in bold, and the related subactivities feed into them. Activities in grey initiate the community nursing service.

Five key worldviews were identified and encapsulated in conceptual models corresponding to the services provided by: specialist children's cardiac centres; local hospitals; GPs; community nursing; health visitors. Figure 2 shows an example of a CATWOE and a Root Definition developed from the perspective of community nursing. These underpin the Activity Diagram for community nursing shown in figure 3, which shows the linked set of purposeful activities required to deliver the service described by the Root Definition. The five main activities are in bold, with related subactivities feeding into each of these. The activities in grey are external prompts that initiate the community nursing service. The full set of Root Definitions and Activity Diagrams are available as online supplementary material.

10.1136/bmjqs-2016-005636.supp1 supplementary methods

### Quantitative OR

**Figure 4:**
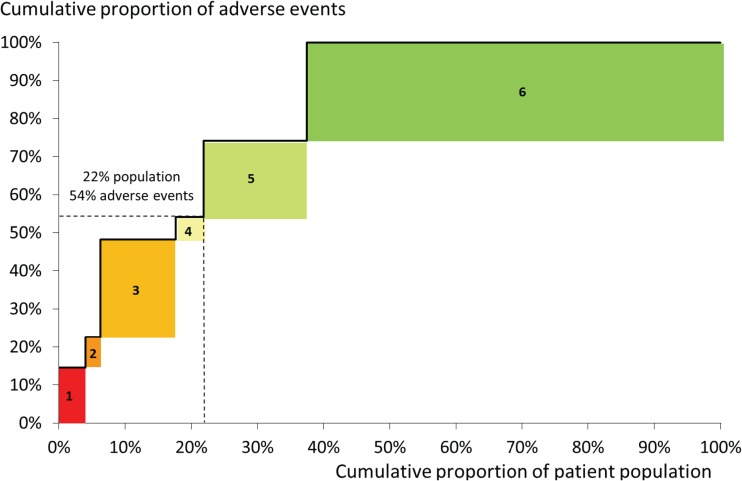
Graphical data summary used to inform prioritisation of service improvements. A graphical data summary developed to inform the decision process of prioritising service improvements. The patient groups were developed using classification and regression tree analysis of linked national UK congenital heart disease and paediatric intensive care audit datasets (see^57^ for details). Groups are ordered from left to right in decreasing risk of adverse events (death or emergency readmission to paediatric intensive care within a year after discharge from infant cardiac surgery). This graph was used in a workshop to guide the Working Group's discussions about targeting interventions at patient groups. When used in the workshop, a detailed key was included to describe the specific clinical features of each group (see^57^ for further details).

Figure 4 shows the graphical data summary that informed the process of prioritising service improvements. It shows the proportion of patients in each of the six groups and the proportion of adverse events among them, with groups ordered from left to right in decreasing risk of adverse events. When used in the workshop, a detailed key showed the combinations of risk factors known at the point of discharge that defined each group (see ref. 57 for further details).

The Working Group used the graph to recommend multidisciplinary care teams only for children with long-term complex needs in addition to their cardiac diagnosis (patient groups 1, 2 and 5). It was also decided that formal home-monitoring programmes (surveillance at home involving saturation and weight monitoring) were only required from a medical perspective for babies with certain high-risk cardiac diagnoses (patient group 3). This was considered a small enough group to make this intervention, expensive at patient level, affordable. Another potential intervention with considerable resource implications was ‘step-down care’ in which patients are discharged from the specialist cardiac centre to their local hospital prior to going home. The Working Group's decision to target this at patient groups 1–4 was informed by recognising from figure 4 that over half of all adverse events were occurring in this 21% of patients.

As an aside, it is worth noting that qualitative evidence from the research study also fed into the decision-making process. For example, it was recommended that non-English speaking families and families with learning difficulties would benefit from more intensive provision of predischarge training and information, because findings from interviews with health professionals suggest they experience greater difficulties in accessing support.^58^ ^59^

### Combined OR

In the final workshop, the operational researcher drew on learning from the Rich Picture and conceptual models (Activity Diagrams) to guide structured decision-making about feasible and acceptable service improvements, and on the data visualisation to facilitate a process of targeting these at patient groups. The qualitative and quantitative OR approaches were therefore both integral to the decision-making process, and complementary in that neither approach could have fulfilled the role of the other. At the end of the workshop, the Working Group had agreed on a coherent set of targeted recommendations for service improvement deemed acceptable from each professional perspective as well as desirable and feasible from a system perspective (see ref. 61 for details of the recommendations themselves).

## Discussion

### Meaning of findings

In our case study, soft systems methodology provided a systematic process to explore the need for, and potential implications of, changes to services for infants with CHD. A Rich Picture was a source of shared learning and engagement for a broad range of stakeholders involved in the complex care pathway. It informed, and was used alongside, conceptual models that the operational researcher used to guide the systematic consideration of potential service change by an expert group. The design and graphical representation of quantitative data analysis helped stakeholders to consider which patient groups to target with which interventions, given resource constraints. The combination of qualitative and quantitative OR methods contributed to the establishment of a coproduced agenda for service improvement.^61^

Study findings (including the Rich Picture and graphical data summary) have been shared with wider interested parties and fed directly into a consultation on standards for CHD services by National Health Service England, and their commissioning of these through service specifications and a Quality Dashboard.^69^ The feasibility of implementing recommendations was a guiding consideration in this work, which we anticipate has enhanced the prospects of uptake. For example, perspectives from all of the services involved regarding issues of feasibility and acceptability were elicited, and notions of prioritisation with respect to limited resources (financial as well as time) were incorporated. The process resulted in a coherent set of endorsed recommendations acceptable to all parties (with acceptability determined via email following the workshop).

### Strengths and weaknesses

The OR techniques described in this article augmented an existing 2-year funded programme of research to which a number of researchers, health professionals and families had already committed their time. This provided a focal point for applying OR iteratively and coherently over time within an existing decision process. In particular, the OR work benefited from evidence that was specifically developed with service improvement in mind during the case study research project.^51–55^ Our conceptual models focused on different worldviews with regard to the service providers, while the Rich Picture was structured around the patient journey and family perspective: the family and their baby are the only people who experience all parts of the system, and so the system-wide perspective that OR brought naturally chimed with a patient-centred view. No non-English speaking families participated in the facilitated workshop, but recommendations relating specifically to non-English speaking families were informed by the qualitative evidence from the case study research project.

The OR approach in this case study was effective in the sense that it had a material impact on the process of developing recommendations. This impact was most tangible in the targeting of recommendations at patient risk groups defined by the quantitative OR analysis. As others have noted,^55^ it is more challenging to isolate the specific influences of problem-structuring methods, as they are complex social interventions. Our own reflection is that, in the absence of an articulated process for considering service improvement systematically in the original research proposal, the SSM was pivotal in providing an overarching framework for doing so. Given the broad endorsement of the ensuing recommendations and their impact on national service standards and commissioning in this area, we infer a degree of effectiveness in the delivery of the SSM framework.

A more comprehensive evaluation of the combined methods would be very instructive. While systematic, there is an element of craft skill involved in conducting the analyses; so, it would also be interesting to probe the extent to which the effectiveness was a result of the OR methods per se, as opposed to personal attributes of the practitioner, the dynamics of the project team or the particular context of the area of application. The roles of these influences may need to be better understood for OR methods to be applied effectively at scale.

We also note that the focus of OR in this research, and our notion of its effectiveness, was its contribution to the robustness of evidence-informed recommendations for service change rather than any downstream impact of the recommendations.

### Implications for policy-makers and clinicians

The combination of qualitative and quantitative OR analyses helped to generate findings of direct practical relevance to clinicians, policy-makers and commissioners regarding a complex service spanning multiple organisations.

OR is a problem-focused discipline in which method selection is guided by the features of the particular problem and decision processes at hand, with flexibility to customise the blend of different quantitative and qualitative approaches. Our case study demonstrates that this warrants further consideration as an approach, for example, by bodies such as the Centre for Clinical Practice at the National Institute for Health and Care Excellence, which is responsible for developing guidance focused on the organisation and delivery of UK healthcare services.

### Future work

The recommendations for improving services for infant CHD included suggestions for further research, implementation and evaluation (see^61^). More broadly, further research is required to understand how and in what circumstances similar combinations of OR methods could be used effectively in other areas of healthcare,^53^ and whether there are viable metrics to assess and inform the support for service change. This could build on recent attempts within the OR literature to develop frameworks for evaluating problem-structuring methods (eg 55) and mixed OR methods within decision support.^50^

Further work to understand how the social sciences (which generate deep understanding) and OR (which tailors understanding to inform decision processes) could be used in a complementary multidisciplinary approach towards quality improvement would also be beneficial.
