# Supplementary material for: Combining qualitative and quantitative operational research methods to inform quality improvement in pathways that span multiple settings
Source: BMJ Qual Saf. 2017 Jan 6;26(8):641–52. doi: 10.1136/bmjqs-2016-005636 (PMC5537516; doi:10.1136/bmjqs-2016-005636)

Online Supplemental Material

The full set of CATWOEs, Root Definitions and Activity Diagrams are set out below for each of the worldviews considered:

- Specialist children's cardiac centres;
- Local hospitals;
- General practitioners (GPs);
- Community nursing;
- Health visitors.

Note that in the Activity Diagrams, activities in grey are considered to initiate the main activities of the service under consideration.

CATWOE and Root Definition for services provided by specialist children's cardiac centres

Customers:

- Infants born with a cardiac malformation that have been discharged from the specialist centre after catheter or surgical intervention, and their families.
- Primary, secondary and community healthcare professionals are also service users in the sense that they need support from specialist centres when specialist knowledge is required and for education/training.

Actors:

- Primarily cardiologists and cardiac specialist nurses (or equivalent).
- They are (sometimes) in contact with: local hospital paediatricians (with/without expertise in cardiology), community nursing teams, health visitors, GPs, social services, psychologists, interpreters, physiotherapists, speech & language specialists and dieticians (based in the specialist centre or local services).

Transformation process

- Babies with a cardiac condition requiring on-going care after discharge receive routine cardiac check-ups and any further cardiac procedures in a timely fashion.
- Families initially knowing nothing about the condition and its implications are given appropriate information and training in order to learn how best to care for their baby, where to go for support and how to recognise and respond to signs and symptoms.
- Relevant local service providers, who initially may be unaware of the baby/family and their needs, receive appropriate referral and are given sufficient patient-specific information and support to care for the babies and families locally, including complex cases that may involve home monitoring.

Worldview

- Cardiac conditions requiring surgery in infancy are rare conditions that need on-going management from specialists
- Some of these babies are particularly vulnerable or have co-morbidities that require additional support services and/or monitoring that can be provided more effectively locally if those providers are sufficiently trained, resourced and informed and if there is effective co-ordination between them and the specialist centre.

Owners:

- Families are either referred through ante-natal screening locally, or referred from their GP/local hospital after the child is born, or through acute emergency admission.
- Congenital heart disease services are a specialised service commissioned directly by NHS England. However the local services such as community nursing and health visitors are funded through generic community services funds commissioned by the Clinical Commissioning Groups.

Environmental constraints:

- Difficulty finding appropriate local services and professionals to contact and not being able to influence the resources available to those services.
- Pressure for beds at specialist centre means discharge is sometimes rushed or there may be bed shortages at the local hospital that prevent planned step-down care (discharge via the local hospital).
- Availability of clinic time at specialist centre or outreach clinics is limited and families sometimes miss appointments.
- There are limited cardiac specialist nursing resources available for setting up co-ordinated care with local services, checking in on families, overseeing monitoring programmes and responding to queries from families and local service providers. Prioritisation of which babies/families are most in need of receiving these resources is required, as well as understanding the provision of services for families who would benefit from this provision but who are not judged to be a high priority.

Root definition: A children's cardiac service delivered predominantly by cardiologists and cardiac specialist nurses (or equivalent) in specialist centres to provide routine cardiac check-ups and any subsequent necessary cardiac procedures for babies. The service also provides the necessary training, information and support to families and local services for them to care for the baby safely at home or as locally as possible.

Activity Diagram for services provided by specialist children's cardiac centres

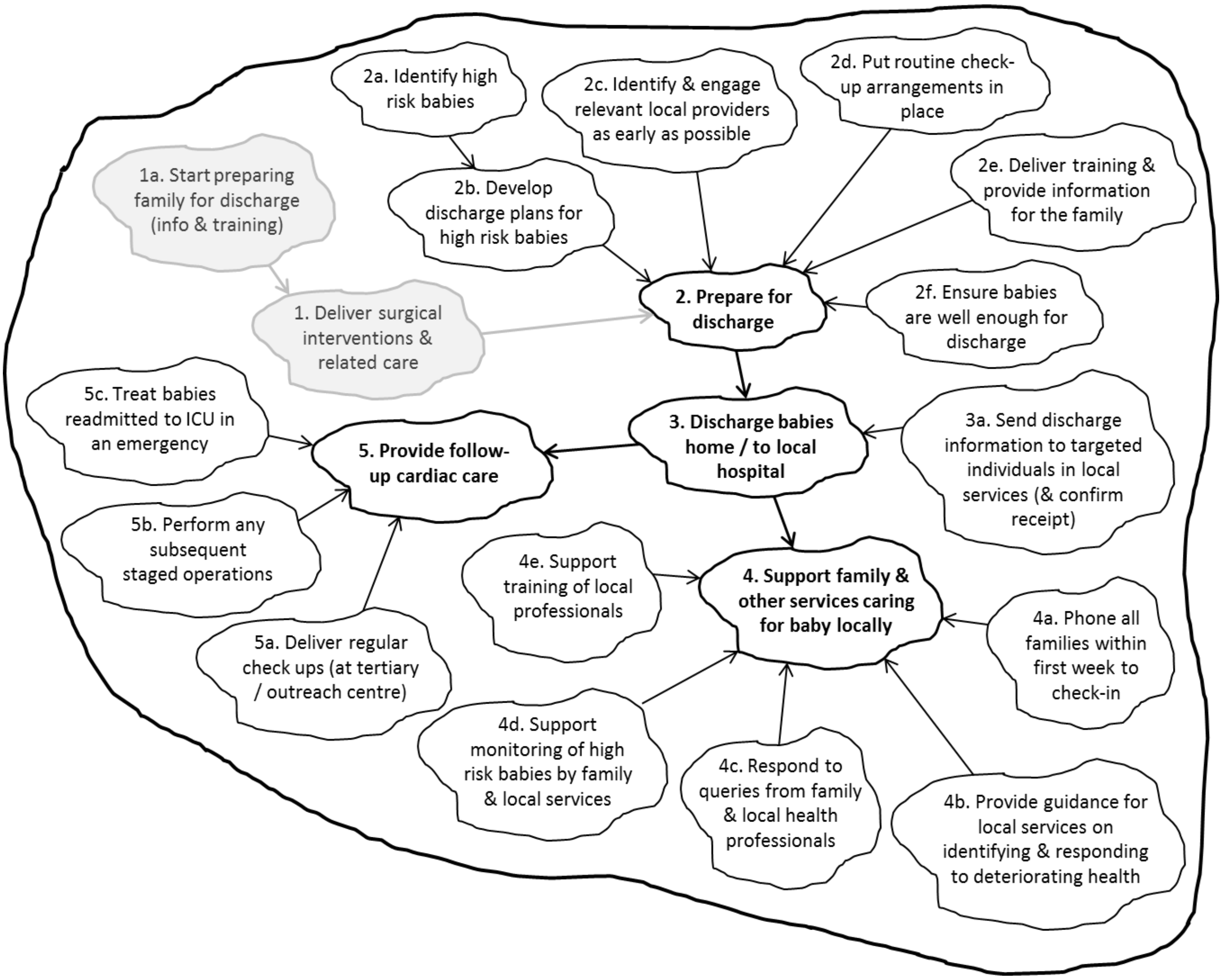

CATWOE and Root Definition for services provided by local hospitals

Customers:

- Cardiac babies that have had intervention, and their families / carers.
- Particularly those babies that are more vulnerable or have feeding difficulties that require step-down care (discharge via the local hospital), or that are likely to experience continuing medical difficulties once home.
- Specialist centre cardiologists sometimes use local hospital facilities to run joint outreach clinics.

Actors:

- General paediatricians and, where available, paediatricians with special expertise in cardiology (PECs).
- They may also be in contact with community nurses, specialist centre cardiologists, cardiac liaison nurses, dieticians, GPs, physiotherapists, psychologists and paediatric pharmacists

Transformation process

- Vulnerable infants that need continuing care at their local hospital prior to discharge home (e.g. in order to gain weight, establish feeding) can be supported locally until they are ready for discharge.
- Infants post cardiac surgery that require regular clinical review are; on a case-by-case basis, assessed nearer home in their local district hospital.
- Families with babies that experience difficulties at home are seen locally by a paediatrician who can refer them back to the specialist centre as required.

Worldview

- It is important for families that their baby receives as much follow-up out-patient care that can be safely be delivered locally, and that their local hospital care is also available if their baby experiences difficulties.
- In particular, it may be preferable that babies who no longer require specialist cardiac care but are not yet ready to go home (e.g. still needing to gain weight) are discharged to the local hospital because it enables local teams to get to know the family/baby and vice versa.
- Locally delivered care can relieve specialist centre service pressure and facilitate patient turnover but there should be quick access to the specialist centre when specialist care is required.

Owners:

- Babies are sometimes, but not always, referred to a named local hospital paediatrician or PEC at discharge from the specialist centre. The responsibility for continuing cardiac and non-cardiac care is defined on a case by case basis and often shared between the two centres. Complex or particularly vulnerable babies may also be referred by the specialist centre for "step-down" care before returning home.
- Clinical Commissioning Groups commission the services provided by local hospitals, although there are no ring-fenced resources specifically for infants with congenital heart disease.

Environmental constraints:

- The workload and availability of PECs (particularly when PECs are on leave/out of hours).
- The level of training and expertise of PECs varies, as does the support available to them.
- The effectiveness of relationships and links the local hospital has with the specialist centres.
- The availability of cardiologists to share clinics and respond to queries the local hospital have.
- The technology for telemedicine or otherwise communicating with specialist centres, and the timeliness and quality of information available to them.
- Resource constraints in accessing interpreters or mobilising social services or other community services (when necessary).
- Issues/constraints also arise around the politics of whose care a patient comes under (specialist or local consultant) and the boundaries of other specialist centres.

Root definition: A hospital run medical service providing local care to babies post-surgery, including regular cardiac clinics, joint outreach clinics, step-down inpatient care for vulnerable children prior to discharge home, medical phone advice and an open access policy in order to ensure that babies are well enough to go home, are treated quickly and locally if their condition deteriorates and receive their routine check-ups close to home

Activity Diagram for services provided by local hospitals

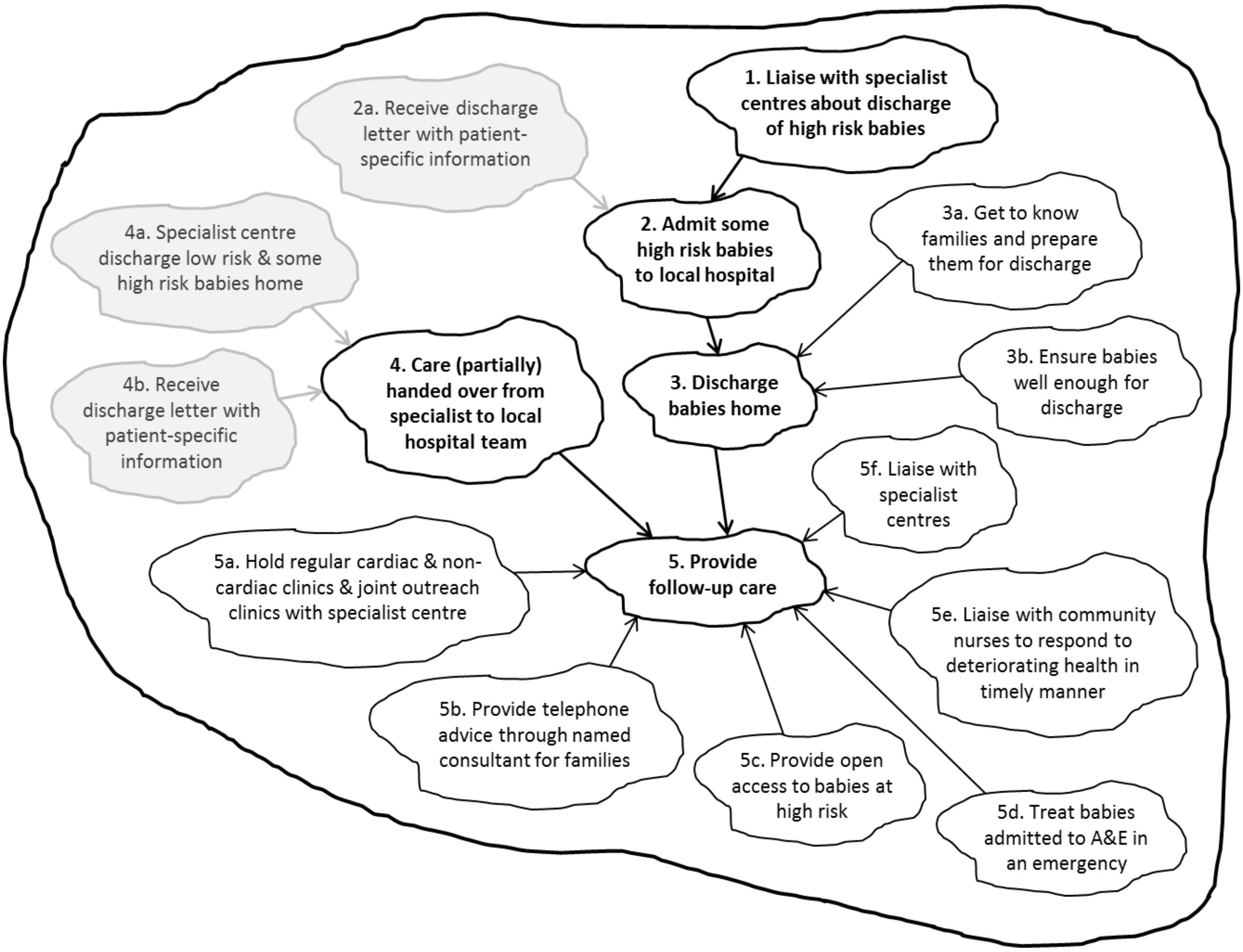

CATWOE and Root Definition for services provided by general practitioners (GPs)

Customers:
- Cardiac babies that have had intervention, and their parents/carers.
- Sometimes other healthcare professionals (such as specialist nurses) use GPs to access information about the child and the community services available for the family.

Actors:
- GPs, who are usually in contact with health visitors and sometimes (e.g. for complex babies) the specialist centre cardiologists and/or specialist nurses, the local hospital (e.g. paediatricians / paediatricians with expertise in cardiology) and community children's nurses.
- If required, they may also be in contact with, or refer on to, social services, mental health services, pharmacists, physiotherapists, speech and language therapists, dieticians and interpreters.

Transformation process
- Babies that potentially require non-urgent, non-cardiac medical care and/or appropriate medications have their needs assessed and met.
- Families seeking advice or psychosocial support have their needs met or are referred to appropriate providers.
- Health professionals who don't know what community services are available for a given family/baby are provided with information and contact details by the GP.

Worldview
- Medication prescriptions and non-urgent non-cardiac medical needs are often best dealt with by the GP, who some families also like to use as a first port of call for seeking psychosocial support or information/advice and who is well placed to refer them on to appropriate services if necessary.

Owners:
- Families contact their GP to arrange appointments.
- Clinical Commissioning Groups and NHS England commission GP services, but there is no specific (ring-fenced) funding for cardiac families.

Environmental constraints:
- GPs are not always contacted by the specialist centre (or local hospital) to notify them that the baby is being discharged and often they first hear about the surgery through the family.
- They often don't receive discharge information in a timely fashion, with discharge letters sometimes taking weeks to arrive by post.
- GPs find that the discharge information they do receive can lack conciseness and contain lots of detailed information regarding the cardiac condition rather than the information that they need, namely: the child's condition, expectations, medication, wound care, signs and symptoms to look out for.
- Sometimes GPs are not fully aware of the range of resources available locally for the baby/family to access.
- Sometimes GPs don't know which other community professionals are already involved in the care of the baby / support of the family and/or don't have access to their contact details.
- GP opening hours can be a constraining factor with families and local hospitals/ambulance staff unable to contact GPs out of hours.

Root definition: An appointment based service run by GP surgeries that provides prescriptions and non-urgent medical care to cardiac babies who require it, a first port of call for families seeking advice and appropriate referral to psychosocial and community services for those families who need it.

Activity Diagram for services provided by general practitioners (GPs)

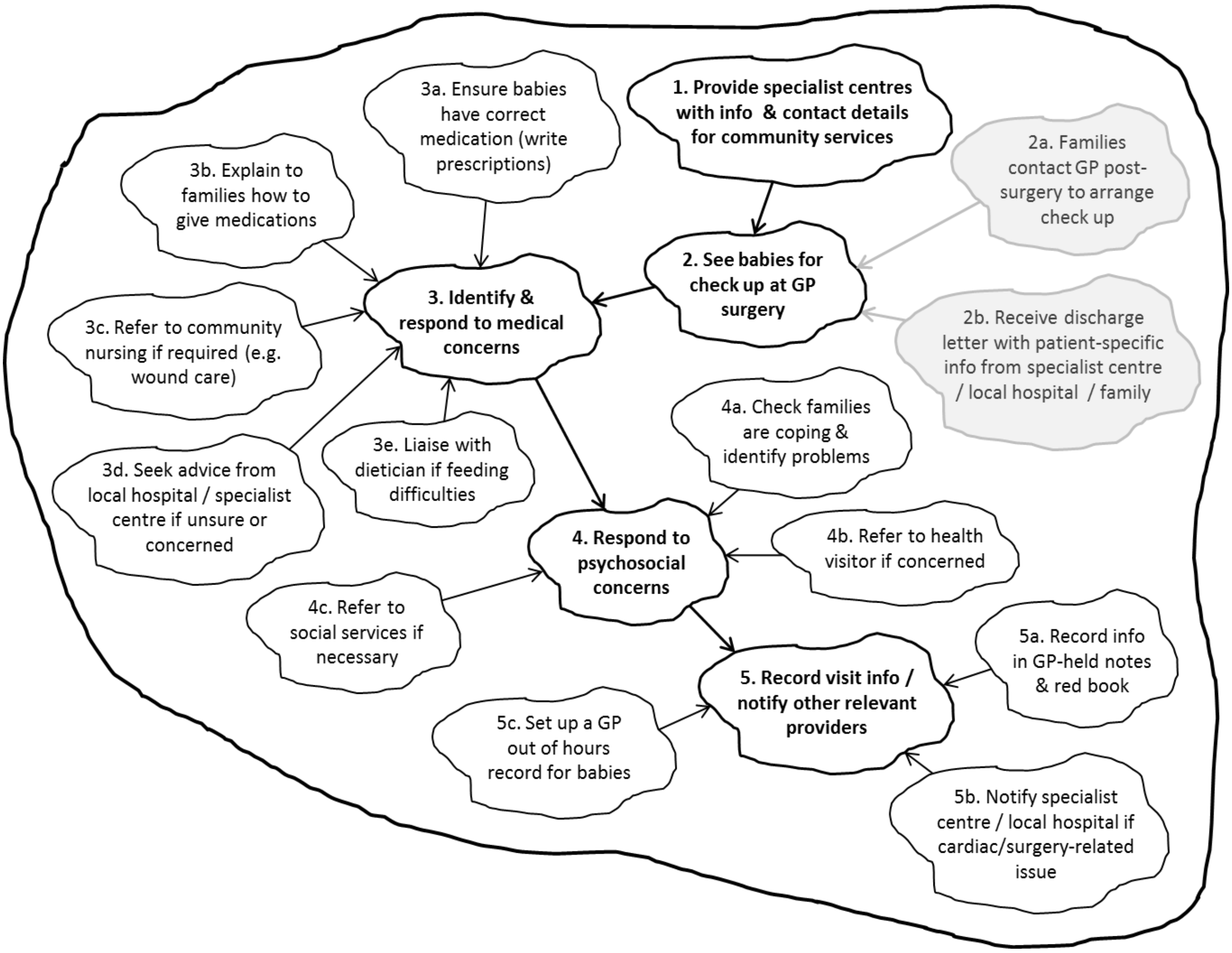

CATWOE and Root Definition for services provided by community nursing

Customers:

- Vulnerable babies with additional medical needs that require attending to in the home and/or their weight and saturation monitored, and their families
- Specialist cardiac centres (that rely on community nurses to alert them to potential deteriorations in a baby's cardiac condition as they can't make regular home visits themselves)

Actors:

- Community nurses, who are sometimes in contact with the local hospital paediatricians (with/without special expertise in cardiology) and the cardiac specialist nurses at the specialist centres
- If the child has complex needs, the community nurses may also be in contact with other community services (e.g. speech and language, physio, and occupational therapists) and, if they have concerns about social issues, social services and/or health visitors

Transformation process

- A baby with additional medical needs post-surgery (e.g. changing nasogastric tube) or requiring inter-stage monitoring between planned procedures has these additional medical needs attended to and any deterioration in their health is spotted and responded to in a timely fashion
- Families demonstrating psychosocial problems that may compromise their ability to care for their baby are flagged and appropriately referred to other services if necessary

Worldview

- Some babies struggle to feed and so in order to gain weight need the support of a community nurse to, for example, change a nasogastric tube and check up on them regularly, whilst others are in a vulnerable medical condition in their inter-stage period (between planned procedures) and need close monitoring so as to prevent sudden deteriorations in their health
- By visiting the family regularly in the home, community nurses are also well placed to notice any psychosocial family problems that are putting the child in danger and raise this concern with other healthcare professionals in a timely fashion

Owners:

- Families are either referred to community nursing at discharge from the specialist centre, or at discharge from the local hospital if they are discharged via there
- Clinical Commissioning Groups commission community nursing services, but not specifically home visits for cardiac babies. The community providers will discharge the patient from their services if there ceases to be a medical reason to be involved

Environmental constraints:

- Specialist centres can have difficulty finding the correct community nurses to contact for a given family (and sometimes there isn't an appropriate service in the family's area)
- Availability of community nurses is constrained by resource pressures and staff shortages: funding is not ring-fenced specifically for cardiac families and community nurses have many other calls on their time
- Community nurses have competing priorities and may not agree with the specialist centre that a cardiac family is of highest priority
- The service that community nurses are able to provide may be limited by the quality of information available to them, in particular how they are supposed to respond to the information they are monitoring and what the expectations for a given baby are
- It may also be limited by the quality of training they receive and the quality of on-going support/link they have with the specialist centre and/or local hospital
- The number of home visits that community nurses are able to make for a given family is restricted by the travel times/distances to visits and their working hours

Root definition: A medical support service delivered in the home by community nurses for babies that have difficulties feeding or other medical needs and/or require home monitoring during their inter-stage period (between planned procedures) in order to help them recover from surgery and to spot and respond to any deterioration in their health whilst they are particularly vulnerable.

Activity Diagram for services provided by community nursing

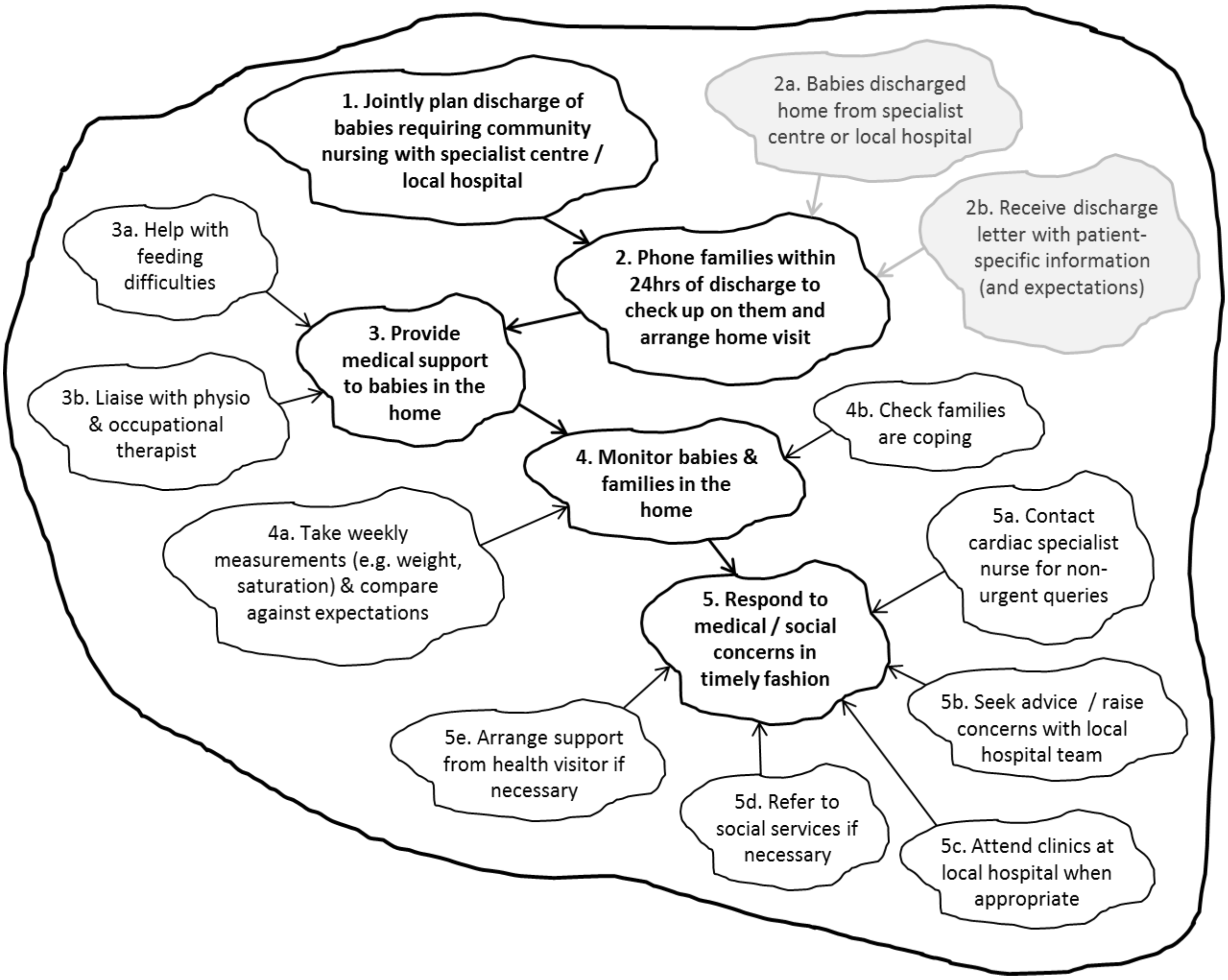

CATWOE and Root Definition for services provided by health visitors

C̲ustomers:

- Primarily parents (rather than the babies).
- The specialist cardiac centres are also service users in the sense that they rely on health visitors to regularly check signs and symptoms in order to be alerted to potential deterioration in a baby's cardiac condition.

A̲ctors:

- Health visitors, who are in contact with General Practitioners (GPs).
- If the child is complex, they may also be in contact with the child development centre and specialist cardiac care team.
- They are in contact with interpreters if required and can contact the paediatric community nurse directly – or sometimes email the paediatrician if they have a specific question.

T̲ransformation process

- Families who are anxious or struggling, want an advocate or need psychosocial support are seen by a health visitor who provides this advocacy and support.
- Specialist centres who are unable to make regular home visits have "eyes and ears" on the ground that can alert them to the deterioration of a baby's health in a timely fashion.

W̲orldview

- Some families are anxious about, or are struggling to, look after their child after surgery and need someone to talk to, provide psychosocial support, act on their behalf and check up on them in order for them to cope better and to alert other services if the child is in danger.

O̲wners:

- Sometimes families are referred to a health visitor at discharge from the specialist centre either directly by the specialist centre or by the GP. Families may also be referred at a later stage by the GP if they feel that psychosocial support is required. However, often the health visitor is not made aware of the cardiac baby and they are informed by the family.
- Clinical Commissioning Groups commission health visitor services. There is no ring-fenced funding specifically for providing these services for cardiac families.

E̲nvironmental constraints:

- Specialist centres can have difficulty finding the correct health visitors to contact for a given family.
- Availability of health visitors is constrained by resource pressures and staff shortages: funding is not ring-fenced specifically for cardiac families and health visitors have many other calls on their time.
- Health visitors have competing priorities and may not agree with the specialist centre that a cardiac family is high priority.
- Health visitors providing the service may be limited by the quality of information available to them (e.g. discharge summaries, signs and symptoms to look for and what to do / who to contact if worried), quality of training they receive and quality of on-going support/link they have with the specialist centre.
- Health visitors providing the service are restricted by travel times/distances to visits and working hours.

Root definition: A non-medical advocacy and psychosocial support service delivered in the community by health visitors to families, if they want it, which are anxious or struggling to look after a baby at home post-surgery in order to help them cope and alert social services or seek medical support if a child is in danger.

Activity Diagram for services provided by health visitors

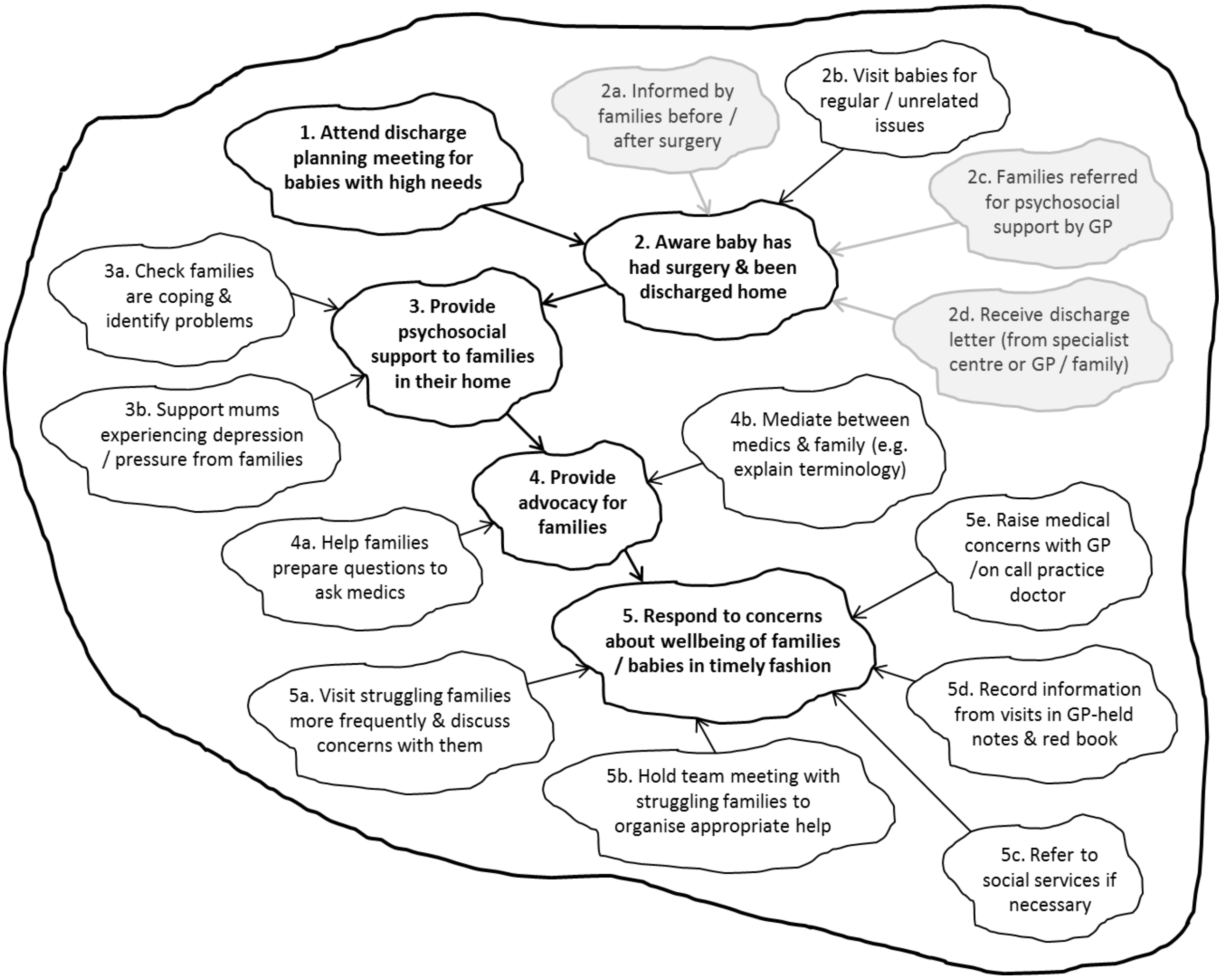

Supplement: supplementary methods [file bmjqs-2016-005636supp001.pdf]
